# Swimming Exercise Pretreatment Attenuates Postoperative Delirium‐Like Behavior in Type 2 Diabetic Rats by Enhancing Mitochondrial Biogenesis Through Activation of SIRT2 Deacetylation

**DOI:** 10.1002/mco2.70142

**Published:** 2025-03-18

**Authors:** Kaixi Liu, Lei Chen, Xinning Mi, Qian Wang, Yitong Li, Jingshu Hong, Xiaoxiao Wang, Yue Li, Yanan Song, Yi Yuan, Jie Wang, Dengyang Han, Taotao Liu, Ning Yang, Xiangyang Guo, Zhengqian Li

**Affiliations:** ^1^ Department of Anesthesiology Peking University Third Hospital Beijing China; ^2^ Research Center of Clinical Epidemiology Peking University Third Hospital Beijing China; ^3^ Department of Anesthesiology Beijing Jishuitan Hospital Capital Medical University Beijing China; ^4^ Department of Anesthesiology and Perioperative Medicine People's Hospital of Zhengzhou University Zhengzhou China; ^5^ Executive Office Beijing Center of Quality Control and Improvement on Clinical Anesthesia Beijing China; ^6^ Perioperative Medicine Branch of China International Exchange and Promotive Association for Medical and Health Care (CPAM) Beijing China; ^7^ State Key Laboratory of Vascular Homeostasis and Remodeling, Department of Anesthesiology, Peking University Third Hospital Beijing China

**Keywords:** mitochondrial biogenesis, postoperative delirium, SIRT2, swimming exercise, Type 2 diabetes mellitus

## Abstract

Postoperative delirium (POD) is a common postsurgical complication that seriously affects patients' prognosis and imposes a heavy burden on families and society. Type 2 diabetes mellitus (T2DM) is a major risk factor for POD. The susceptibility mechanisms of POD in T2DM individuals and the role of exercise preconditioning remain unclear. Adult rats with and without T2DM were used to assess the promotive effect of diabetes on postoperative delirium‐like behavior. The diabetic rats were also subjected to a swimming exercise program before surgery. The potential beneficial effect of exercise preconditioning on postoperative cognition was evaluated by examining neurobehavior, hippocampal neuroinflammation, mitochondrial morphology, and function in diabetic rats. Finally, underlying mechanisms were further analyzed by exploring the role of the sirtuin family in vivo and in vitro. We found that performing tibial fracture surgery resulted in delirium‐like behavior and inhibited hippocampal mitochondrial biogenesis in diabetic rats but not in healthy rats. Preoperative swimming exercise was beneficial in attenuating delirium‐like behavior, inhibiting neuroinflammation, and improving mitochondrial biogenesis and function. Preoperative swimming exercise achieved these positive effects by upregulating SIRT2‐mediated peroxisome proliferator‐activated receptor gamma coactivator‐1 alpha (PGC‐1α) deacetylation and activating mitochondrial biogenesis in T2DM rats.

## Graphical abstract

The reduction of SIRT2 following orthopedic surgery in the hippocampus of T2DM rats inhibits the acetylation process of PGC‐1α and the subsequent mitochondrial biogenesis process, leading to mitochondrial dysfunction and postoperative delirium‐like behavior. Preoperative swimming exercise upregulates SIRT2 and PGC‐1α expression in hippocampal neurons, activating mitochondrial biogenesis, preserving mitochondrial function, and improving postoperative cognitive outcomes in T2DM rats.

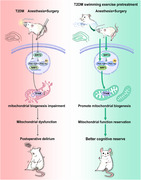

## 1 Introduction

Postoperative delirium (POD) is an enigmatic postoperative complication characterized by inattention and acute alterations in the level of consciousness and cognitive functioning, leading to both short‐ and long‐term postoperative complications and a 2‐ to 20‐fold increase in postoperative mortality risk [1, 2]. Risk factors for POD vary depending on the type of surgery and perioperative management, including aging, multiple comorbidities, medication use, and preoperative cognitive dysfunction [3]. Type 2 diabetes mellitus (T2DM), a prevalent chronic disease, is identified as an independent risk factor for POD. Given the rising volume of surgical procedures, the POD‐related healthcare burden on cognitively vulnerable T2DM patients is increasing [4, 5]. The global prevalence of T2DM is projected to increase by 51% to 783 million cases by 2045 [4]. The escalating incidence of diabetes in the elderly population is widely acknowledged. T2DM in young adults leads to lifetime adverse effects and unfavorable complications, raising the possibility of a future public health catastrophe [6]. Dementia is more likely to develop in patients with diabetes by 50%–140%, and cognitive decline is increased by 20% to 50% in patients without dementia. Moreover, middle‐aged and older diabetic patients are 26% more likely to develop postoperative cognitive dysfunction [7]. The dramatic increase in T2DM prevalence has led to growing concern regarding T2DM‐related postoperative cognitive dysfunction.

The primary purported mechanisms of POD include neuroinflammation, oxidative stress, neurotransmitter imbalance, and sleep–wake cycle disruption [8, 9, 10]. Nevertheless, the precise mechanism of action remains unclear. Mitochondria, serving as essential organelles for intracellular energy generation, are crucial in preserving neural function. Anesthesia and surgery have been implicated in the pathogenesis of POD by enhancing oxidative stress through epigenetic modulation of hippocampal mitochondrial DNA (mtDNA) methylation, which mediates mitochondrial structural and functional damage and leads to mitochondrial dysfunction in POD‐related studies [1, 11]; the mechanism underlying mitochondria's role in the elevated risk of POD development in individuals with T2DM is still unclear.

Exercise has long been considered a cornerstone of treatment for T2DM [12]. Mitochondria are critical organelles through which exercise improves neuronal plasticity and adaptation, thus playing a crucial role in maintaining normal neural function. However, even though exercise contributes to perioperative brain health, it is difficult for patients to fulfill “exercise prescriptions” in the perioperative period. Therefore, investigating the molecular mechanisms through which exercise helps ameliorate postoperative neurological injury is expected to lead to the development of alternative interventions. Swimming is a widely recognized aerobic exercise for rodents, delivering comprehensive health benefits with minimal joint impact. This makes it an ideal, consistent intervention for exploring the physiological effects of exercise. Previous studies indicated that the modulation of mitochondrial function by sirtuins serves as a key mechanism underlying the cognitive improvement associated with exercise. Mammalian sirtuins, a group of conserved proteins (Sirtuin 1–7) serving as key regulators of energy metabolism through their activities as nicotinamide adenine dinucleotide (NAD^+^)‐dependent deacetylase and ADP‐ribosyltransferase activities, play a crucial role in modulating various stress response pathways [13]. Sirtuin 2 (SIRT2) deficiency in primary hippocampal neurons leads to mitochondrial fragmentation and autophagy damage, and brain tissue from SIRT2‐deficient mice exhibits mitochondrial dysfunction [14]. SIRT1 and SIRT3 also help regulate the mitochondrial biogenesis process in neurodegenerative diseases [15, 16]. NAD^+^ accumulating during exercise can act as a substrate for sirtuins, upregulate sirtuin expression through a positive feedback mechanism, increase hippocampal brain‐derived neurotrophic factor expression, inhibit hippocampal apoptosis and inflammatory responses, and activate the autophagy lysosomal system [17, 18, 19]. Clinical studies have also shown that resistance exercise increases serum SIRT1, SIRT3, SIRT5, and SIRT6 levels [20, 21]. However, there is no relevant report on the impact of exercise interventions on POD prevention and control via modulation of sirtuin‐mediated processes. Given the crucial role of deacetylation modification of the sirtuins in exercise adaptation and mitochondrial function, we further hypothesized that exercise could break the vicious cycle of mitochondrial dysfunction and neuroinflammation associated with sirtuins in the postoperative period and ultimately reduce susceptibility to POD.

Our study explored whether T2DM increases the risk of POD‐like behavior and whether exercise before surgery can offer neuroprotective benefits to diabetic rats. Furthermore, we delved into the underlying mechanisms of the possible protective effects by examining neuroinflammation and mitochondrial health in the hippocampus. Specifically, we hypothesized that exercise preconditioning could improve mitochondrial dysfunction after surgery by regulating sirtuins and affecting downstream deacetylation modification.

## 2 Results

### 2.1 Delirium‐Like Behavior and Neuroinflammation Were Induced by Tibial Surgery in T2DM Rats

**FIGURE 1:**
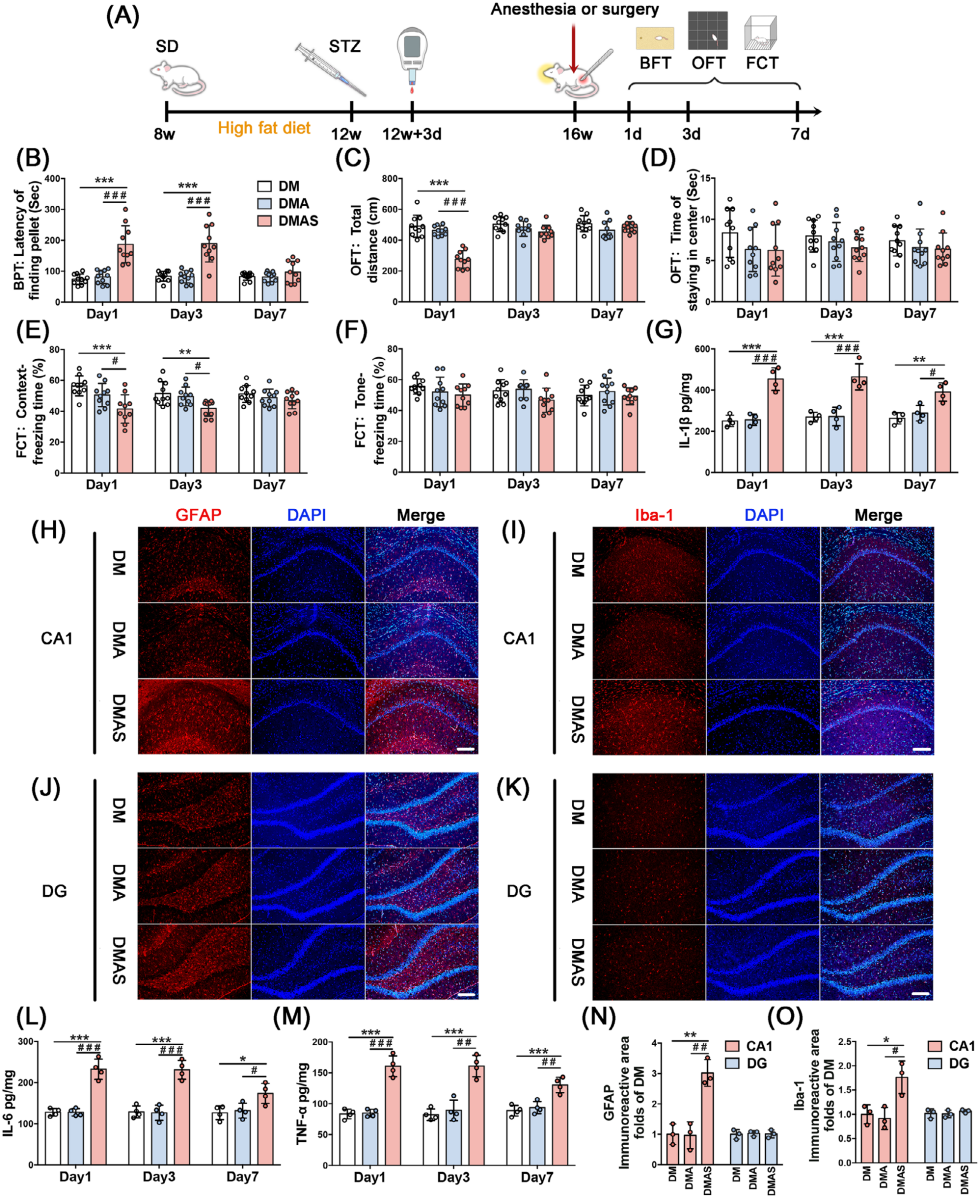
Effect of anesthesia or surgery on postoperative neurobehavior and neuroinflammatory response in diabetic rats. (A) Schematic representing the chronological order of T2DM modeling, tibial fracture surgery, and behavioral testing. (B) T2DM rats receive BPT on Postoperative Days 1, 3, and 7. (C) Total distance and (D) the time of staying in the center of T2DM rats in OFT after anesthesia or tibial fracture surgery. (E) Freezing time of context test and (F) tone test of T2DM rats in FCT after anesthesia or tibial fracture surgery. (G, L, M) Expression levels of pro‐inflammatory factors in hippocampal tissue of T2DM rats. (H–K) Expression of GFAP and Iba‐1 in hippocampal CA1 and DG regions, scale bars = 100 µm. (N, O) Statistics of GFAP and Iba‐1 fluorescence area in CA1 and DG regions. *p < 0.05, **p < 0.01, ***p < 0.001 compared with DM group. ^#^ *p* < 0.05, ^##^ *p* < 0.01, ^###^ *p* < 0.001 compared with the DMA group.

We found that normal rats who received 30 min of sevoflurane anesthesia or tibial surgery did not exhibit altered behavior (Figure S1A–E). Regarding T2DM rats, significantly increased latency to find pellets was observed at 1 and 3 days after tibial surgery (Figure 1B). On the third day after surgery, comparable movement capacity among diabetic groups suggested that the poorer performance (Figure 1C) in the DMAS group resulted from impaired natural behavior or olfactory function.

On Days 1 and 3 following tibial surgery, freezing time was significantly reduced in the context test. A decrease in freezing time was not observed in the DMAS group during the tone test (Figure 1E,F). Thus, only hippocampal‐dependent spatial memory was impaired in the DMAS group. Moreover, 30 min of sevoflurane anesthesia did not cause any behavioral changes in normal adult or T2DM rats. Hence, our results suggest that orthopedic surgery plus anesthesia could induce delirium‐like behavior in T2DM rats but not in normal adult rats.

On Days 1, 3, and 7 postoperatively, hippocampal samples were collected to determine pro‐inflammatory cytokine levels. On Day 1 post‐surgery, the levels of interleukin‐1β (IL‐1β), interleukin‐6 (IL‐6), and tumor necrosis factor‐α (TNF‐α) were significantly increased in surgical groups compared with nonsurgical groups. On Postoperative Day 3, the DMAS group had a higher level of neuroinflammation, which persisted until 7 days after tibial surgery. Furthermore, 30 min of sevoflurane anesthesia alone did not induce any changes in pro‐inflammatory cytokines in normal (Figure S1F–H) or T2DM rats (Figure 1G,L,M).

Tibial surgery induced significant morphological changes in astrocytes and microglia in the hippocampus, which displayed amoeba‐like morphology following tibial surgery. GFAP and Iba‐1 expression levels were significantly increased in the DMAS group, but there were no significant changes in the DMA group (Figure 1H–K,N,O) on Day 3 post‐surgery.

### 2.2 Mitochondrial Dysfunction Was Induced in the Hippocampus of T2DM Rats After Tibial Surgery

**FIGURE 2:**
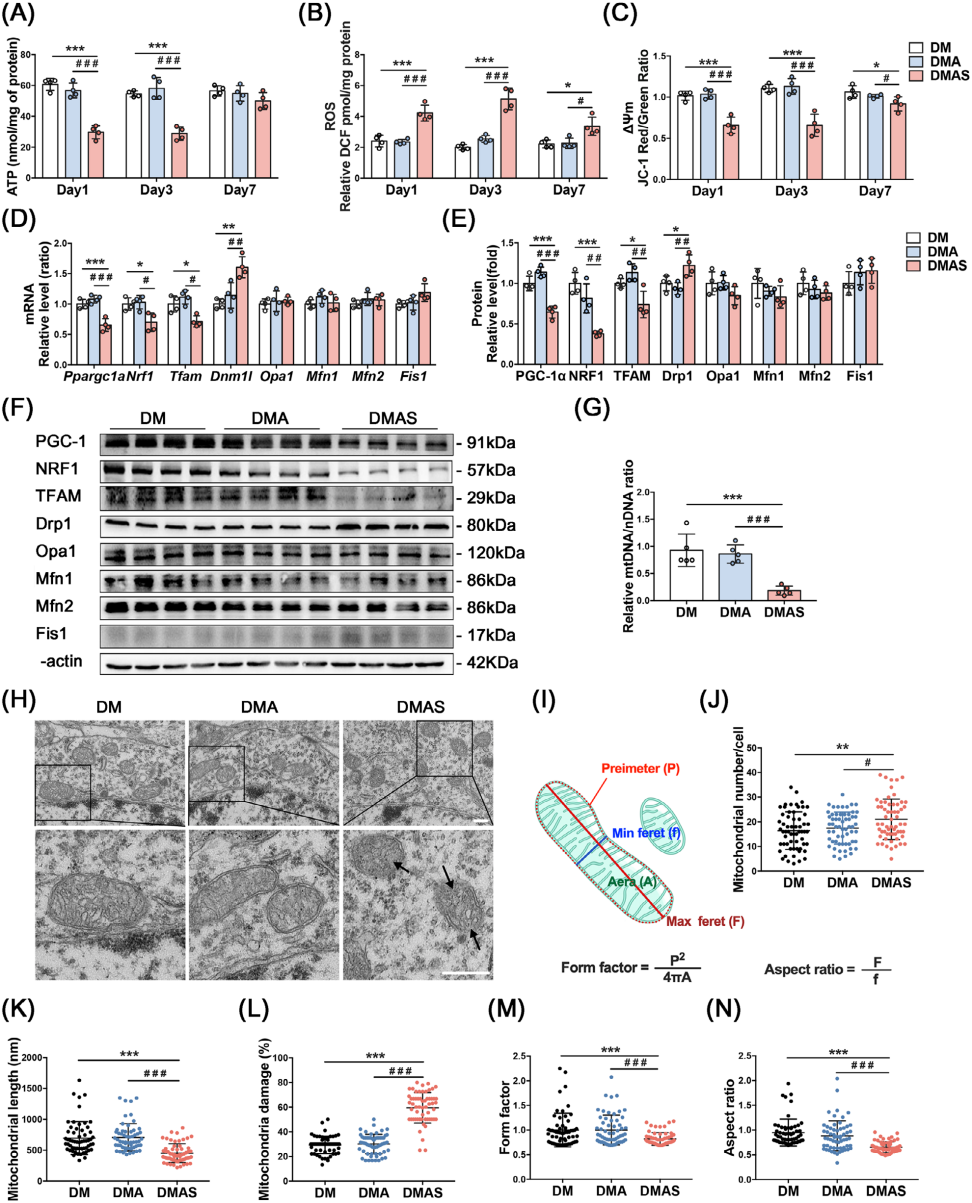
Effect of anesthesia or surgery on mitochondrial function in diabetic rats. (A–C) Effect of anesthesia or surgery on ATP, ROS, and MMP levels in the hippocampus of diabetic rats. (D) Relative mRNA expression levels of mitochondrial biogenesis and dynamics‐related molecules. (E) Statistical analyses and (F) representative bands of mitochondrial biogenesis and dynamics‐related molecules in the hippocampus. (G) Relative levels of mtDNA/nDNA. (H) Mitochondrial morphology in the hippocampus, scale bars = 500 nm. (I) Schematic diagram of mitochondrial circumference, major diameter, and minor diameter, calculation formulas for form factor and aspect ratio. (J) Mitochondrial number, (K) length, (L) damage percentage. (M) Mitochondrial form factor and (N) aspect ratio (*n* = 3 rats per group, 20 cells per rat). *p < 0.05, **p < 0.01, ***p < 0.001 compared with the DM group. ^#^ *p* < 0.05, ^##^ *p* < 0.01, ^###^ *p* < 0.001 compared with the DMA group.

In the hippocampal tissue of normal adult rats, there were no significant changes in the ATP content or ΔΨ_m_ level (Figure S1I–K), except that the reactive oxygen species (ROS) level in the ConAS group was higher than that in the Con group on the first day after surgery (Figure S1J). Meanwhile, T2DM rats showed a significant decrease in ATP production and the ΔΨ_m_ level, along with a significant increase in ROS production, the ΔΨ_m_ level, and a significant increase in ROS production after tibial surgery. Moreover, the reduced ΔΨ_m_ level and high ROS production in the hippocampus of diabetic rats persisted until 7 days after surgery (Figure 2A–C).

Hippocampal protein and mRNA levels of molecules related to mitochondrial biogenesis and dynamics of normal adult rats that underwent tibial fracture surgery were not significantly altered compared with those of the nonsurgical group (Figure S1L,N,O), and there were no statistically significant group differences in mtDNA/nuclear DNA (nDNA) (Figure S1M). mtDNA/nDNA in the DMAS group showed a significant reduction compared with nonsurgical groups. However, 30 min of sevoflurane anesthesia did not induce significant changes (Figure 2G). In T2DM rats, the mRNA levels of all mitochondrial biogenesis‐related proteins were significantly reduced in the hippocampus after tibial surgery compared with the nonsurgical group, where the levels of each protein indicate mitochondrial biogenic dysfunction (Figure 2D–F).

In normal adult rats, mitochondrial function was sufficient to overcome surgical stress (Figure S1I–K), whereas in T2DM rats, impaired mitochondrial function exacerbated dysfunction after surgical stress. A significant increase in mitochondrial number and damage, along with a decrease in length (Figure 2J–L), occurred in the DMAS group. Fragmented mitochondria with vacuoles and no cristae structure indicated mitochondrial damage after tibial surgery in T2DM rats. A more detailed analysis of single mitochondria was then performed using transmission electron microscopy (TEM) images, and form factor (FF) and aspect ratio (AR) values were calculated (Figure 2I). FF has a value of 1 when the mitochondria form a perfect circle. A value of FF > 1 indicates that mitochondria are longer and have more complex shapes [22]. AR is the ratio between the major and minor axes of the mitochondrial ellipse, indicating elongation and fragmentation of the mitochondria. After tibial surgery, there was a significant reduction in the FF and AR in the DMAS group compared with the nonsurgical group (Figure 2M,N).

### 2.3 Swimming Exercise Attenuated Delirium‐Like Behavior and Enhanced Mitochondrial Function Following Tibial Surgery in T2DM Rats

**FIGURE 3:**
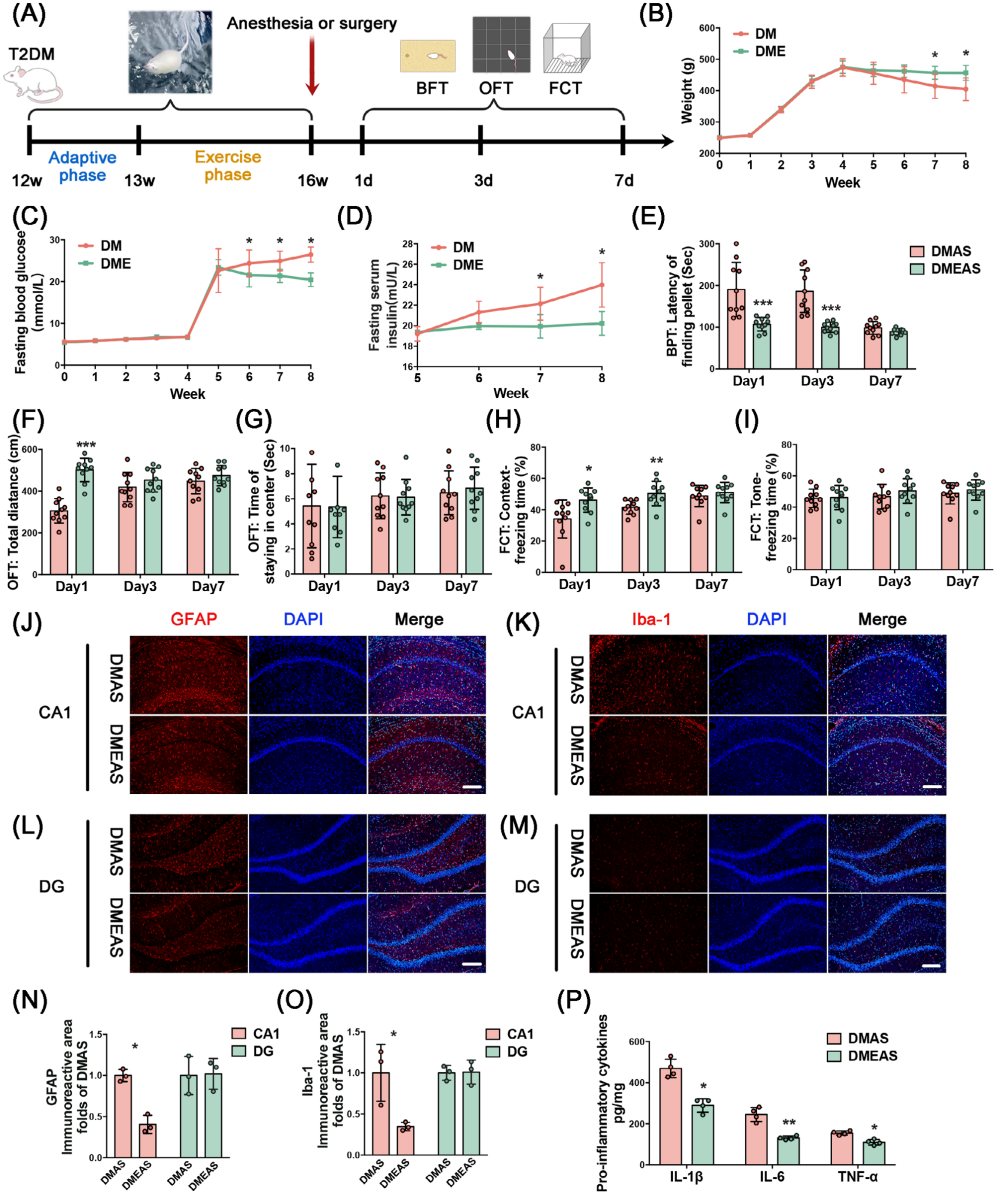
Swimming exercise pretreatment ameliorates delirium‐like behavior and hippocampal neuroinflammatory response after surgery in T2DM rats. (A) Schematic representing chronological order of swimming exercise pretreatment, tibial fracture surgery, and behavioral testing. (B) Trends in body weight, (C) fasting blood glucose, and (D) fasting serum insulin in non‐exercised and exercise‐pretreated rats. (E) Latency of finding a pellet in BPT, (F) total distance, (G) the time of staying in the center in OFT, (H) freezing time of the context test, and (I) tone test in FCT on Postoperative Days 1, 3, and 7. (J–M) Expression levels of GFAP and Iba‐1 in hippocampal CA1 and DG regions, scale bars = 100 µm. (N, O) Statistics of GFAP and Iba‐1 fluorescence area in CA1 and DG regions. (P) Expression levels of pro‐inflammatory factors in hippocampal tissue. *p < 0.05, **p < 0.01, ***p < 0.001 compared with the DMAS group.

Four weeks of swimming exercise alleviated insulin resistance and lipoatrophy in T2DM rats. Specifically, the fasting serum insulin and fasting glucose levels of the DMEAS group significantly decreased, whereas body weight increased, compared with the sedative group (Figure 3B–D). T2DM rats that completed 4 weeks of swimming exercise displayed improvement in POD‐like behavior. The latency to find pellets was significantly decreased (Figure 3E), and the freezing time in the context test was significantly increased (Figure 3H) in T2DM rats that engaged in swimming exercise compared with sedated rats. In the CA1 region of T2DM rats, the immunoreactive areas of GFAP and Iba1 were reduced after swimming exercise in comparison with sedentary rats following tibial surgery (Figure 3J–O). On the third postoperative day, the levels of pro‐inflammatory cytokines were also significantly reduced after exercise pretreatment (Figure 3P).

**FIGURE 4:**
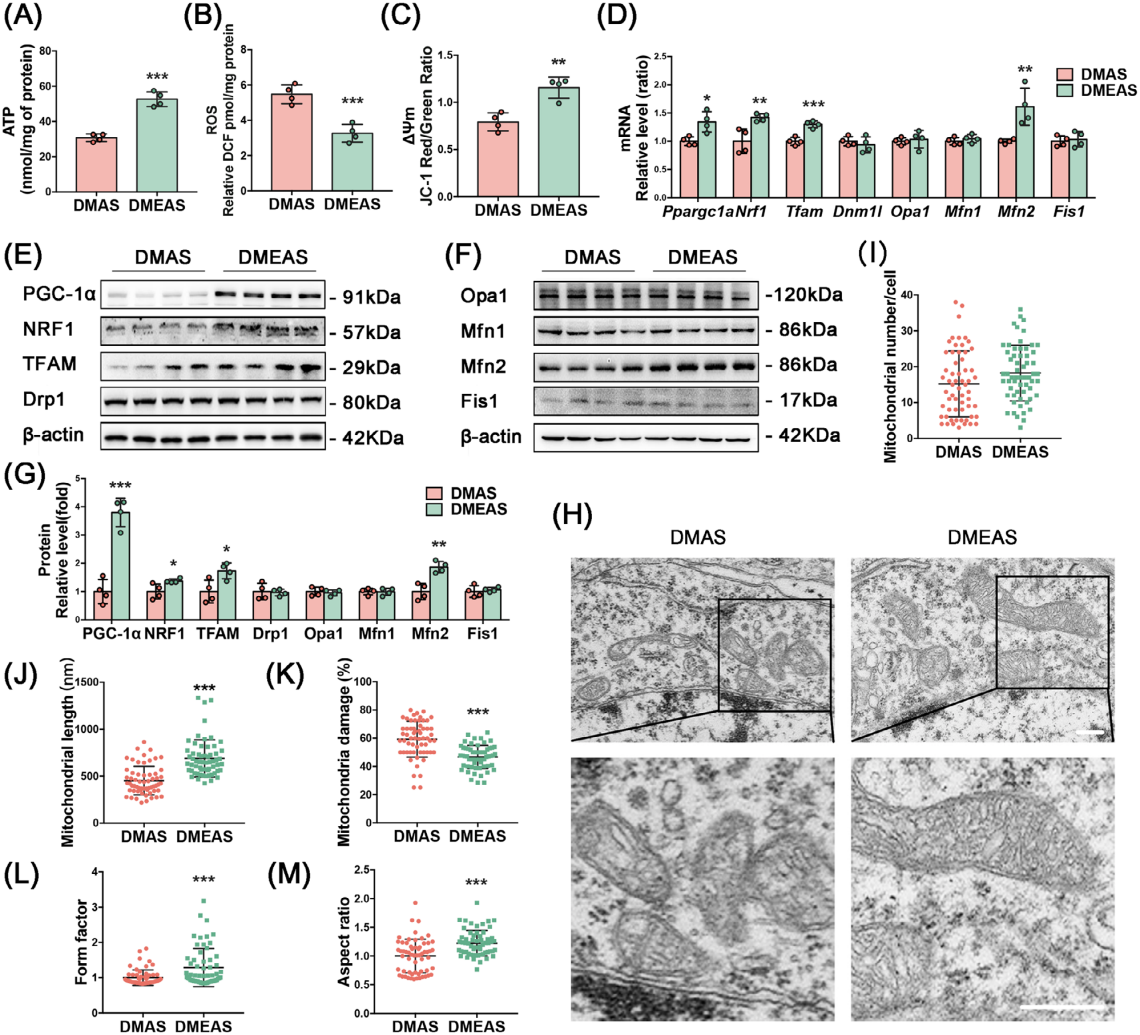
Swimming exercise pretreatment attenuates postoperative hippocampal mitochondrial dysfunction in T2DM rats. (A, B) ATP, ROS, and MMP levels in the hippocampus of non‐exercised and swimming exercise pretreatment T2DM rats. (D) Relative mRNA expression levels of mitochondrial biogenesis and dynamics‐related molecules. (E, F) Representative bands and (G) statistical analyses of mitochondrial biogenesis and dynamics‐related molecules in the hippocampus. (H) Mitochondrial morphology in the hippocampus, scale bars = 500 nm. (I) Mitochondrial number, (J) length, (K) damage percentage, (L) form factor, and (M) aspect ratio (*n* = 3 rats per group, 20 cells per rat). *p < 0.05, **p < 0.01, ***p < 0.001 compared with the DMAS group.

T2DM rats that engaged in swimming exercise showed a significant increase in ATP production and ΔΨ_m_ levels, as well as a significant decrease in ROS levels compared with those without exercise (Figure 4A–C). Polymerase chain reaction (PCR) results showed that *Ppargc1a*, *Nrf1*, and *Tfam* expression levels were significantly upregulated in the exercise group compared with the sedative group (Figure 4D). Consistently, WB analysis confirmed that the protein levels of PGC‐1α, NRF1, and TFAM were significantly increased in the DMEAS group compared with the DMAS group (Figure 4E–G). TEM showed that swimming induced a significant increase in mitochondrial length and cristae structure (Figure 4H). After exercise and surgical stress, the mitochondrial number did not change significantly, but mitochondrial fragmentation and damage percentage were reduced, the length was extended, and mitochondrial cristae were clearer than in the DMAS group (Figure 4I–K). Further, swimming pretreatment improved the FF and AR, indicating better‐preserved mitochondrial morphology and elongation of the mitochondrial structure (Figure 4L,M).

### 2.4 SIRT2 Deficiency Reversed Exercise‐Mediated Cognitive Improvement and Mitochondrial Function in T2DM Rats Following Tibial Surgery

**FIGURE 5:**
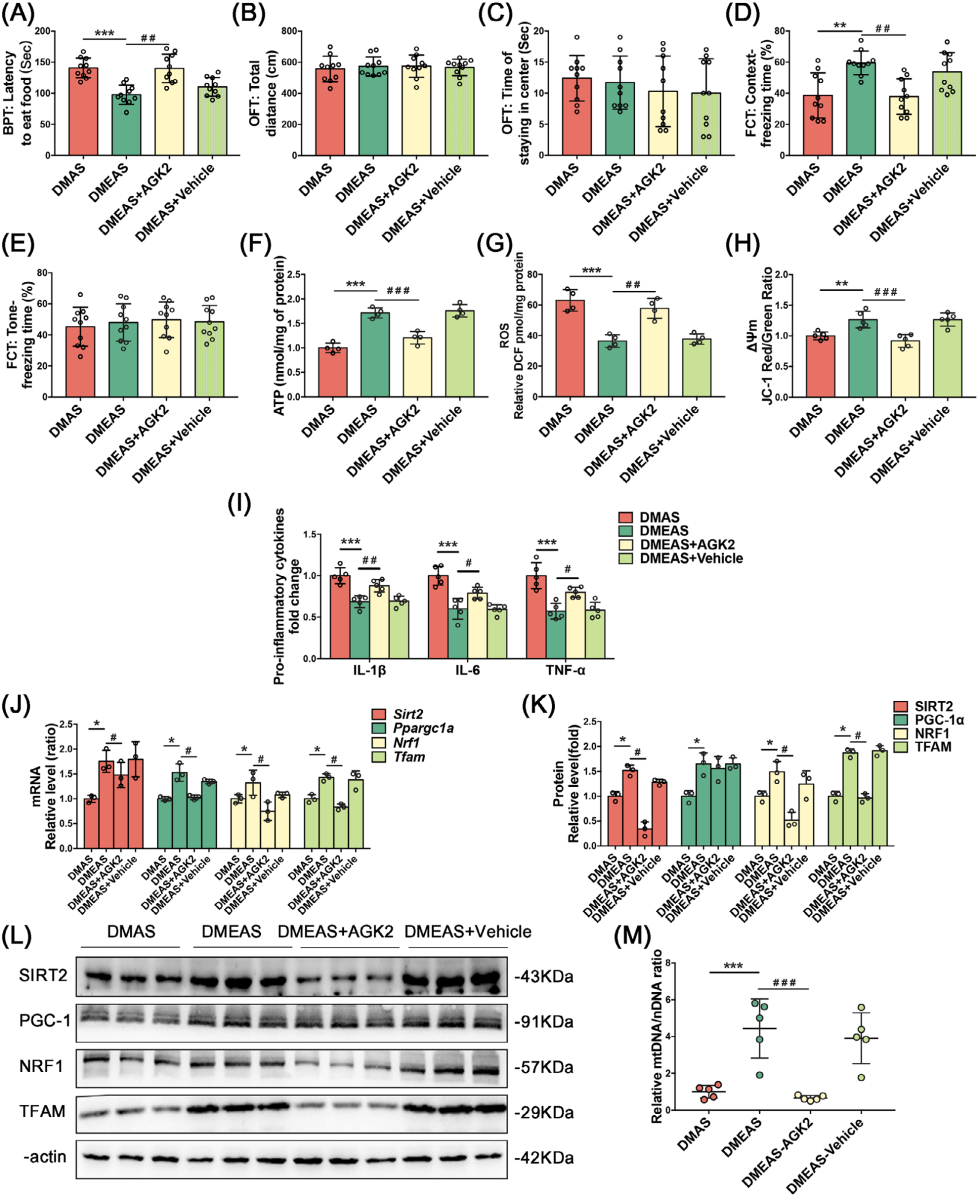
AGK2 brain area injection weakened the ameliorating effect of swimming exercise pretreatment on postoperative delirium‐like behavior and mitochondrial function in T2DM rats. (A) Latency of finding a pellet in BPT, (B) total distance, (C) the time of staying in the center in OFT, (D) freezing time of the context test, and (E) tone test in FCT on Postoperative Day 3. (F–H) ATP, ROS, and MMP levels in the hippocampus. (I) Expression levels of pro‐inflammatory factors in hippocampal tissue on Postoperative Day 3. (J) Relative mRNA expression levels of *Sirt2* and mitochondrial biogenesis‐related molecules. (K) Statistical analyses and (L) representative bands of SIRT2 and mitochondrial biogenesis‐related molecules in the hippocampus. (M) Relative levels of mtDNA/nDNA. *p < 0.05, **p < 0.01, ***p < 0.001 compared with the DMAS group. ^#^ *p* < 0.05, ^##^ *p* < 0.01, ^###^ *p* < 0.001 compared with the DMEAS group.

SIRT2 was screened as the target molecule that may ameliorate POD‐like behavior in T2DM rats (Figure S2A). To explore whether the ameliorative effect of swimming exercise pretreatment is dependent on the effect of SIRT2 on mitochondrial biogenesis‐related proteins, we delivered a SIRT2‐specific inhibitor, AGK2 (IC₅₀ = 3.5 µM), via stereotactic injections into the CA1 hippocampal brain of T2DM rats to downregulate SIRT2 following swimming exercise. The DMEAS‐AGK2 group took longer to find pellets and had shorter freezing times in the context test (Figure 5A–E). These results suggest that inhibition of SIRT2 expression in the CA1 region of the hippocampus suppressed the ameliorative effect of swimming exercise pretreatment on POD‐like behavior in T2DM rats.

Mitochondrial function results revealed decreased ATP content and ΔΨ_m_ levels and increased ROS production in hippocampal tissue in the DMEAS‐AGK2 group compared with the DMEAS group (Figure 5F–H). ELISA showed that the injection of solvents into the CA1 hippocampal area did not significantly change the levels of the pro‐inflammatory factors (Figure 5I). The PCR results showed that the expression of *Ppargc1a*, *Nrf1*, and *Tfam* was significantly reduced in the DMEAS‐AGK2 group compared with the DMEAS group (Figure 5J). WB showed that the expression of SIRT2, NRF1, and TFAM was downregulated in the DMEAS‐AGK2 group compared with the DMEAS group, whereas the expression of PGC‐1α was not significantly altered (Figure 5K,L). The mtDNA/nDNA ratio in the DMEAS‐AGK2 group was considerably lower than in the DMEAS group (Figure 5M).

### 2.5 Overexpression of SIRT2 Ameliorated High Glucose Plus Conditioned Culture Media‐Induced Impairment of Neuronal Mitochondrial Biogenesis

**FIGURE 6:**
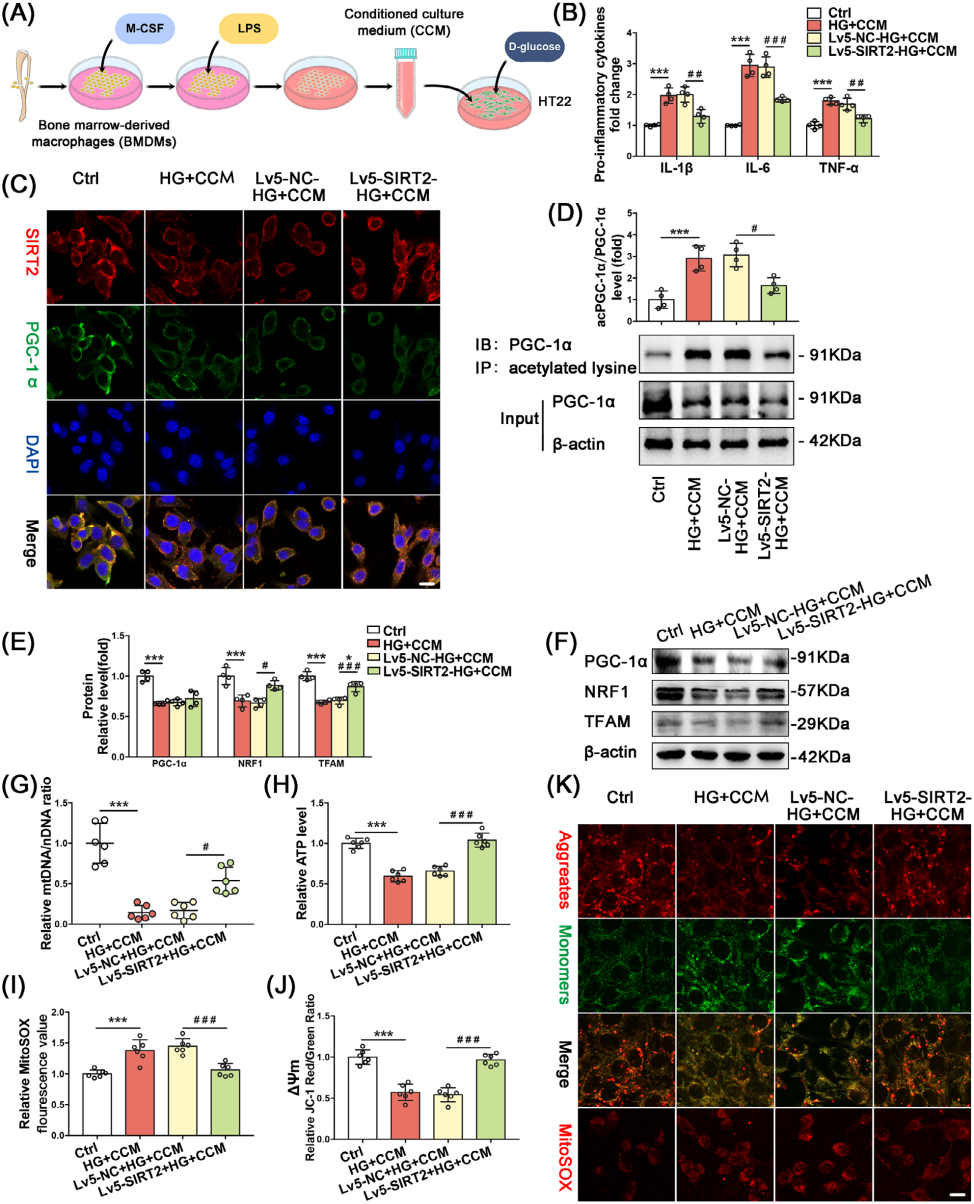
Overexpression of SIRT2 improves HG + CCM‐induced mitochondrial dysfunction by upregulating PGC‐1α deacetylation level to ameliorate HG + CCM‐induced mitochondrial dysfunction. (A) Conditioned medium extraction flowchart. (B) Relative expression levels of pro‐inflammatory factors in HT22 cells. (C) IF detection of SIRT2 and PGC‐1α expression and co‐localization in HT22 hippocampal neurons, scale bars = 10 µm. (D) Representative bands and statistics of acetylated PGC‐1α expression detected by immunoprecipitation assay. (E) Statistical analyses and (F) representative bands of mitochondrial biogenesis‐related molecules in HT22 cells. (G) Relative levels of mtDNA/nDNA. (H) Relative levels of ATP production, (I) mitoSOX, and (J) MMP. (K) Representative images of JC‐1 aggregates/monomers and mitoSOX expression in HT22 cells, scale bars = 10 µm. *p < 0.05, **p < 0.01, ***p < 0.001 compared with the Ctrl group. ^#^ *p* < 0.05, ^##^ *p* < 0.01, ^###^ *p* < 0.001 compared with the Lv5‐NC‐HG+CCM group.

The extraction process of conditioned culture media (CCM) and the optimal stimulation conditions for HT22 cells were described in Supporting Information. ELISA of pro‐inflammatory factors showed that the levels of IL‐1β, IL‐6, and TNF‐α were significantly higher in the high glucose plus conditioned medium (HG+CCM) groups compared with the normal medium (Ctrl) groups. The expression levels of IL‐1β, IL‐6, and TNF‐α were significantly lower in the Lv5‐SIRT2‐HG+CCM group compared with the HG+CCM group (Figure 6B). The protein expression of NRF1 and TFAM was significantly increased in the Lv5‐SIRT2‐HG+CCM group compared with the HG+CCM group, while PGC‐1α expression was not significantly different between the groups (Figure 6E,F). The mtDNA/nDNA levels were substantially lower in the HG+CCM group than in the Ctrl group, whereas the mtDNA/nDNA levels were significantly higher in the Lv5‐SIRT2‐HG+CCM group compared with the HG+CCM group (Figure 6G).

### 2.6 SIRT2 Ameliorated Neuronal Mitochondrial Biogenesis Dysfunction by Upregulating PGC‐1α Deacetylation

In HT22 cell lines, immunofluorescence results showed that SIRT2 and PGC‐1α were significantly colocalized in the cytoplasm, neuronal SIRT2 and PGC‐1α expression was downregulated in the HG+CCM treated group, and PGC‐1α expression was not significantly altered in the Lv5‐SIRT2‐HG+CM group. These results were consistent with those of WB (Figure 6C). Immunoprecipitation was applied to detect the effect of overexpression of SIRT2 on the acetylation level of PGC‐1α in the HG+CCM group. The ratio between total acetylated PGC‐1α in IB and total PGC‐1α in Input showed that the total acetylation level of PGC‐1α in the Lv5‐SIRT2‐HG+CCM group was significantly reduced compared with that in the HG+CCM group (Figure 6D).

The mitochondrial function results showed that ATP content and MMP significantly decreased, whereas MitoSOX levels increased dramatically in the HG+CCM group compared with the Ctrl group. Moreover, ATP content and MMP were increased, whereas MitoSOX levels were decreased in the Lv5‐SIRT2‐HG+CCM group compared with the HG+CCM group (Figure 6H–J). The mitochondrial function results are shown in fluorescence images of JC‐1 monomer/polymer and MitoSOX; the representative fluorescence images were consistent with the functional assay results (Figure 6K).

## 3 Discussion

Delirium is characterized by an acute change in mental status that fluctuates over time. Symptoms include inattention, disorganized thinking, and altered levels of consciousness. Recent research on POD using modeling and several neurobehavioral tests has shown promise in identifying neuroinflammatory markers [1, 11, 23]. In our study, rats were selected for modeling diabetes and delirium‐like behaviors, as well as for swimming exercise intervention; due to their physical advantages, tibial fractures were done to simulate clinical orthopedic surgery. Using a food reward experiment as a clinical attention test, Velagapudi et al. found that attention deficit was most severe 24 h after surgery in adult mice. However, complete recovery was seen on Postoperative Day 5, indicating a valid endpoint for detecting delirium‐like behavior [24]. In this study, compared with the nonsurgical group, T2DM rats on Postoperative Day 3 returned to baseline in total distance traveled in the OFT but had a significantly prolonged food‐finding latency, indicating abnormalities in natural behaviors and inattentiveness. Additionally, a significant reduction in freezing time in the context of the fear conditioning test (FCT) reflected disorganized thinking and impaired learning abilities. These findings suggested that delirium‐like behavioral alterations persisted at this time. As the different models develop delirium‐like behaviors at different time points in mice [25, 26, 27, 28, 29], the time points of neurobehavioral testing we used in rats were consistent with Wang et al.’s research [30].

Our previous research showed that T2DM is the major risk factor for POD in orthopedic surgery patients [31], and the in vivo experiments in this study further confirmed that T2DM facilitates the development of POD‐like behavior. T2DM is associated with processes such as decreased mitochondrial function, endoplasmic reticulum stress, and impaired energy metabolism, such that the vicious cycle of mitochondrial dysfunction and neuroinflammation may be the key mechanism underlying increased POD susceptibility in individuals with T2DM. Surgery‐induced tissue trauma can trigger an immune response leading to neuroinflammation. This response increases the production of pro‐inflammatory cytokines in the serum and the hippocampus, disrupts the blood‐brain barrier, and promotes inflammation [32, 33]. In HT22 cells, decreased mitochondrial function and respiratory capacity were detected after 1.5 h of TNF‐α exposure [34]. In our study, the significantly higher levels of pro‐inflammatory factors, together with significant mitochondrial dysfunction, in the DMAS group corroborated the vicious cycle mentioned previously. In our in vitro study, HT22 cells were stimulated in the HG+CM group to mimic the effects of inflammatory mediators of peripheral origin on neuronal mitochondrial function in diabetic hyperglycemia. Similar to other research, pro‐inflammatory factor expression and mitochondrial function also decreased significantly, and the expression of mitochondrial biogenesis‐related molecules significantly declined under diabetic conditions [33, 35, 36].

PGC‐1α plays a crucial role in regulating mitochondrial biogenesis and energy metabolism. Inflammatory factors alter mitochondrial function by regulating microRNA expression within the mitochondrial complex and inhibiting the transcriptional cofactor PGC‐1α, thereby reducing mitochondrial biogenesis [37]. Several studies have shown that pro‐inflammatory factors could lead to downregulation of protein and transcript levels of PGC‐1α [38, 39, 40], and the hippocampal tissues in our DMAS group also showed significantly lower PGC‐1α expression and mitochondrial function compared with the nonsurgical groups. However, this was improved by exercise pretreatment. In HT22 cells, we further demonstrated that neuronal pro‐inflammatory factor levels were significantly reduced by upregulating SIRT2‐mediated deacetylation of PGC‐1α and activating the mitochondrial biogenesis pathway under high‐glucose and inflammatory conditions, suggesting that both attenuating the inflammatory response and boosting the functional reserve of mitochondria could play a key role in combating POD.

Anesthesia and surgery disrupt mitochondrial dynamic processes in the hippocampus of aged mice, leading to excessive mitochondrial division and inducing delirium‐like behavioral changes [41]. Similarly, our results showed that T2DM rats undergoing surgery showed delirium‐like behavior, decreased expression of mitochondrial biogenesis‐related molecules, increased expression of Drp1, and mitochondrial morphology characterized by fragmentation with vacuole formation in the hippocampus. Additionally, we established anesthesia‐only groups to investigate the independent effect of anesthesia on T2DM rats. The results indicated that short‐term anesthesia did not result in significant mitochondrial damage or delirium‐like behavior. Exercise can contribute to a more robust mitochondrial phenotype and restore mitochondrial homeostatic balance, thereby resisting the adverse effects of anesthetic surgery. Mitochondrial function was significantly superior in the postoperative hippocampus in our DMEAS group compared with the non‐exercise group, and mitochondrial morphology was characterized by less damage, indicating exercise acclimatization and enhancement of mitochondrial functional reserve.

This study found that SIRT2 levels were closely associated with delirium‐like behavior. Sirtuins could alleviate surgery‐induced mitochondrial dysfunction and delirium‐like behavior by modulating mtDNA methyltransferase activity [1], and we hypothesized that fluctuations in SIRT2 influence susceptibility to delirium‐like behavior in T2DM rats. In the cortex and hippocampus of mammals, SIRT2 is widely distributed, functioning as a deacetylase and being the only protein primarily found in the cytoplasm that can also move between the mitochondria and nucleus [42]. Consistent with our results, SIRT2 deficiency caused mitochondrial dysfunction and increased oxidative stress via elevated protein acetylation and decreased ATP levels [14, 43, 44], in turn enhancing the function of SIRT2 in the central nervous system [45, 46]. Controlling diet and energy intake can upregulate SIRT2; exercise, an energy‐consuming metabolic process, may have a similar effect [47]. Exercise pretreatment promoted the upregulation of SIRT2 and PGC‐1α in this study, but PGC‐1α expression after stereotactic injection of AGK2 and lentiviral expression of neuronal SIRT2 were unchanged. Instead of affecting total PGC‐1α expression, the absence of SIRT2 may prevent acetylated PGC‐1α from entering the nucleus, thereby hindering the activation of mitochondrial biogenic pathways and leading to mitochondrial dysfunction and delirium‐like behavior in T2DM rats. Similarly, Liu et al. found that the expression of PGC‐1α was identical between brain tissues of wild‐type and Sirt2^−/−^ mice, but acetylated PGC‐1α was significantly increased, and mitochondrial morphology in embryonic fibroblasts was altered in Sirt2^−/−^ mice, resulting in swollen and cristae‐sparse mitochondria [14]. Upregulation of SIRT2 by exercise pretreatment or overexpression of neuronal SIRT2 can improve mitochondrial biogenesis and dysfunction. The regulatory role of SIRT2 provides a new perspective on the prevention and treatment of POD in highly susceptible individuals unable to fulfill the “exercise prescription.”

The present study had several limitations. First, only male rats were used in this study, and we cannot exclude the effect of genetic factors on behavioral outcomes and physiological states. Second, we only conducted a 4‐week voluntary swimming exercise protocol. The effects and underlying mechanisms of other forms and protocols of exercise on delirium‐like behavior remain to be explored. Third, the role of SIRT2 on mitochondrial function was only explored in neurons; its effect on other cell types remains to be verified. In addition to its role in mitochondrial biogenesis, the role of SIRT2 in regulating other processes of mitochondrial homeostasis warrants further study.

## 4 Conclusion

T2DM promotes POD‐like behaviors in rats, accompanied by decreased hippocampal SIRT2 expression and mitochondrial biogenesis. Preoperative swimming exercise improves postoperative mitochondrial function and attenuates POD‐like behavioral changes by upregulating hippocampal SIRT2 expression, promoting deacetylation of PGC‐1α in hippocampal neurons, and activating the mitochondrial biogenesis pathway in T2DM rats.

## 5 Material and Methods

### 5.1 Animals

Animal Ethics Committee approval was obtained (LA2021041), and the study was conducted following the ARRIVE 2.0 guidelines for the Care and Use of Animals in Research [48]. Adult male Sprague Dawley rats (weight: 200 ± 20 g) were purchased from Huafukang Bioscience Co. Ltd. (Beijing, China). Group‐housed (three per cage) rats were maintained at room temperature (24 ± 1°C), with humidity of 50%–60% and free access to food and water. Rats were given 1 week to acclimate to their housing conditions before the experiment began.

Induction of T2DM in rats was achieved using a high‐fat diet (HFD) and low‐dose streptozotocin (STZ) [49]. Three groups of rats were randomly divided after 1 week: a control group (*n* = 60), an HFD group (*n* = 90), and an HFD + exercise group (*n* = 60). The rats in the HFD group were fed an HFD (D12492, Research Diets Inc., New Brunswick, NJ, USA) for 4 weeks. In the fourth week of HFD, rats were fasted for 12 h with free access to water, and each rat was injected intraperitoneally with 30 mg/kg STZ (Sigma‐Alrich, St. Louis, MO, USA). A regular diet was fed to rats in the control group, and the same dosage of citrate buffer was injected. Blood glucose levels were measured 3 days after STZ injection from the tail vein. Further investigations were conducted on rats with random blood glucose > 16.7 mmol/L in the HFD group [50]. A total of 160 rats were used for T2DM modeling, with a success rate of 93.75%, and 150 rats ultimately met the criteria for successful induction. Rats that failed to meet the diagnostic criteria after injection were excluded from the study.

### 5.2 Swimming Exercise

We trained T2DM rats five times a week in a swimming pool (160 cm diameter, 50 cm height) filled with water at 30 ± 1°C. The swimming program included an adaptive phase and an exercise phase. To decrease water‐induced stress, swimming exercises of gradually increasing duration (10, 20, 30, 40, and 50 min/day) allowed for acclimation in the adaptive phase. Three 20‐min swimming sessions were followed by a 10‐min rest period. Animals were dried with towels after swimming and kept warm with electric heaters for 15 min afterward.

### 5.3 Surgical Trauma

We performed an open tibial fracture with intramedullary fixation of the left hind paw in aseptic surgical conditions, with a modification of the anesthesia protocol [51]. Animals were given a general anesthetic (3% sevoflurane in 0.30 FiO_2_). Shaving and disinfection were performed on the rats' left tibias. Following a median incision on the lower two‐thirds of the tibia, a 20‐G pin was inserted into the intramedullary canal. A periosteal stripping and osteotomy were then performed. After producing a tibial fracture, the wound was irrigated, and the skin was sutured with 8/0 Prolene sutures. A warming pad was used to maintain the temperature while the animals spontaneously recovered from the anesthetic. The total duration of anesthesia and surgery was fixed at 30 min, a 2% lidocaine solution was applied locally before the incision, and 1% a tetracaine hydrochloride mucilage was applied to the wound twice daily until Day 3.

### 5.4 Behavioral Tests

POD is characterized by acute concurrent disturbances at diverse cognitive levels affecting natural and learned behaviors [52]. Animal models of delirium have mainly been used to test natural and learned behaviors [10, 27, 53]. The buried pellet test (BPT) [11], open field test (OFT) [54], and FCT [55] were used to assess POD. The BPT and OFT assess changes in the natural behavior of rats, while the FCT assesses changes in learned behavior.

Using the BPT, rats were assessed for their natural tendency to locate olfactory cues [56]. Three pieces of sweet cereal were given to each rat 2 days before the test. Rats were habituated to the testing environment for at least 1 h before being tested in their home cages. During habituation, the test cage (48 cm length × 25 cm width × 20 cm height) was prepared with 5‐cm‐deep clean bedding, and a piece of sweetened cereal was randomly buried 3.8 cm below the surface of the bedding. After the rat had been in the cage for 5 min, its latency to eat the food was measured as the time it took for it to uncover the pellet and grasp it in its forepaws or teeth. A latency of 300 s was recorded if the rat failed to find the pellet within 5 min.

After the BPT and 30 min before the FCT, the OFT was conducted. Under dim lighting, rats were gently placed in an open field chamber and allowed to move around freely for 5 min. Using SMART 3.0.1 (San Diego Instruments, San Diego, CA, USA), movement parameters were automatically calculated. Rat locomotor activity and anxiety are reflected in the total distance traveled and time spent in the central zone. To prevent the presence of olfactory cues, the chamber floor was cleaned with 75% ethanol after each test.

There are two phases to the FCT test: a training phase before surgery and a test phase after surgery at 1, 3, and 7 days. The rats underwent fear conditioning training 1 day before surgery, which involved being placed in a conditioning chamber for 180 s and exposed to three pairs of conditional–unconditional stimuli, followed by an additional 60 s in the chamber. The pairs of conditional stimuli consisted of a 60 s, 80 dB sine wave tone (conditional stimulus) and a 1 s, 0.8 mA electrical foot shock. FCT tests hippocampal‐dependent memory in the context test and hippocampal‐independent memory in the tone test [57]. Rats were simply placed back into the conditioning chamber without any tones or foot shocks during the context test. During the tone test, the rats were placed in a novel chamber with a different painting for 5 min, followed by 3 min of tone delivery. A tracking software recorded the percentage of rat freezing time.

### 5.5 Levels of Inflammatory Factors

Hippocampal levels of the pro‐inflammatory markers IL‐1β, IL‐6, and TNF‐α were quantified via enzyme‐linked immunosorbent assay (ELISA) kits (Neobioscience, Shenzhen, China), following the guidelines provided by the manufacturer. Results are expressed as pg of cytokine per mg of tissue.

### 5.6 Western Blot

Using RIPA buffer containing phosphatase and protease inhibitors, the hippocampus and cells were lysed. Following the extraction of proteins, the supernatants were centrifuged for 15 min at 4°C and quantified with the BCA protein assay kit (Beyotime Biotechnology, Shanghai, China). Proteins were separated by 10% sodium dodecyl sulfate–polyacrylamide gel electrophoresis and transferred onto polyvinylidene difluoride membranes in equal amounts. 5% fetal bovine serum (FBS) in Tris‐buffered saline containing 0.1% Tween 20 was used to block the membranes, and the following primary antibodies were incubated overnight at 4°C: anti‐PGC‐1α (Abcam, ab106814), anti‐NRF1 (Abcam, ab175932), anti‐TFAM (Abcam, ab272885), anti‐Drp1 (Santa Cruz, sc‐271583), anti‐Fis1 (Santa Cruz, sc‐376447), anti‐OPA1 (Santa Cruz, sc‐393296), anti‐Mfn1 (Santa Cruz, sc‐166644), anti‐Mfn2 (Santa Cruz, sc‐515647), anti‐SIRT2 (Abcam, ab211033), and anti‐β‐actin (Santa Cruz, sc‐47778). Secondary antibodies were incubated for 1 h at room temperature, followed by an enhancement of chemiluminescence and subsequent analysis with Gel‐Pro software (Media Cybernetics, Rockville, MD, USA).

### 5.7 Quantitative Real‐Time PCR

With the help of the TRIzol reagent, total RNA was extracted from the hippocampus or cells, and complementary DNA was synthesized with a reverse transcription kit (Invitrogen, Carlsbad, CA, USA). An ultraviolet spectrophotometer was used to measure total RNA concentration (Nanodrop ND‐100, Thermo Fisher Scientific, Wilmington, DE, USA). A260/A280 values all fell between 1.8 and 2.0, indicating high purity. Shenggong Biotech Co. Ltd. (Shanghai, China) designed and synthesized the primers. PCR was performed using SYBR Green qPCR Master Mix (Tiangen, Beijing, China). Expression levels were normalized to *Actb* levels and calculated using the formula 2^−ΔΔ^ *^C^* ^t^.

### 5.8 Measurement of mtDNA Copy Number

We extracted genomic DNA from hippocampal tissue and HT22 cells using the DNeasy Blood & Tissue Kit (Qiagen, Shanghai, China) as directed by the manufacturer [58]. A real‐time quantitative PCR was used to determine the copy number of mtDNA/nDNA. The ABI Prism 7500HT system (Applied Biosystems, Waltham, MA, USA) was used to perform real‐time quantitative PCR experiments with TB Green Premix Ex Taq II (TAKARA, Shiga, Japan). The primers used to amplify mtDNA and nDNA were listed in Supplementary [3, 59–61]. The relative mtDNA copy number was calculated with the 2^−ΔΔ^ *^C^* ^t^ method [62].

### 5.9 Immunofluorescence

Transcardial injections of phosphate‐buffered saline (PBS, pH 7.3) and 4% paraformaldehyde were administered to rats. The tissues were embedded in OCT, and 10 µM sections were prepared. Iba‐1 (Merck Millipore, 019–19741) and GFAP (CST, 3670S) expression levels were investigated to elucidate the activation of microglia and astrocytes. Antibodies against GFAP and Iba‐1 were incubated with sections, followed by 1 h of secondary antibody incubation with Alexa Fluor CY3 (Jackson, 111‐165‐003, 115‐165‐003). Images of the slides were then taken (Leica Microsystems, Wetzlar, Germany).

### 5.10 Adenosine Triphosphate Measurement

An ATP assay kit (Beyotime Biotechnology) was used to measure hippocampal ATP. Each sample was tested for protein concentration using a BCA protein assay kit (Beyotime Biotechnology). Amounts of ATP are expressed as nmol/mg protein.

### 5.11 ROS Measurement

ROS levels were quantified by dichlorodihydrofluorescein diacetate (DCFH‐DA) [63]. Using cool Locke's buffer at 1:20, hippocampal homogenates were diluted to 2.5 mg tissue/500 µL. Lock's buffer (1 mL, pH 7.4) and 200 mL of homogenate were mixed in the reaction mixture, and 10 mL of DCFH‐DA (5 mM) was incubated at room temperature for 15 min, followed by analysis with a spectrofluorometer (Promega, Madison, WI, USA) with excitation at 484 nm and emission at 530 nm. Parallel blanks were used to measure background fluorescence. The expression of ROS was quantified on a histogram.

### 5.12 Isolation of Mitochondria

The mitochondrial fraction was isolated using the Qproteome Mitochondria Isolation Kit (Solarbio Science & Technology Co., Beijing, China). Fresh mitochondria were used for membrane potential detection.

### 5.13 Measurement of Mitochondrial Membrane Potential

By staining hippocampal tissue with JC‐1, we quantified the red/green ratio. A mitochondrial extraction kit (Solarbio) was used to extract the mitochondria from hippocampal tissue. The MMP of mitochondria in each group was determined using an MMP assay kit (Beyotime Biotechnology) with JC‐1 according to the manufacturer's instructions. The isolated mitochondria suspension was incubated with 1.5 µM of the JC‐1 dye for 30 min at 37°C, rinsed twice with PBS, and then detected with a fluorescence spectrophotometer (F‐4500, Hitachi, Tokyo, Japan). The red/green fluorescence intensity ratio was used to determine MMP.

### 5.14 Transmission Electron Microscopy

TEM was performed [64]. Briefly, rats were perfused with PBS and injected with a mixed fixative solution (25% glutaraldehyde and 0.2 M PBS). Hippocampal tissue was cut into 1 mm^3^ pieces and fixed in 2.5% glutaraldehyde for 24 h, followed by 1% osmic acid for 2 h. The tissue was then dehydrated with acetone and embedded in epoxy. Using an ultramicrotome, ultrathin slices (70–80 nm) were cut, and hippocampal mitochondria were examined using JEOL JEM‐1400 at 1000× magnification.

### 5.15 Stereotaxic Injection

Sevoflurane was used to anesthetize the rats, which were then positioned in an animal stereotaxic apparatus (RWD Life Science, Shenzhen, China). The skull hole was made with a microsurgical drill and injected with a 5 µL Hamilton syringe (Hamilton, Bonaduz, Switzerland). Drug administration was controlled by a micro syringe pump controller (RWD Life Science, Shenzhen, China), and the syringe was pulled out 10 min after drug administration. All drugs were injected using the exact stereotaxic coordinates (measured from bregma: −3.96 mm anteroposterior, ±2.5 mm mediolateral, and −3.15 mm dorsoventral). SIRT2 inhibitor AGK2 (MedChemExpress, HY‐100578) was dissolved in 4% dimethyl sulfoxide and diluted in PBS. The volume of AGK2 (200 µM) was 2.5 µL per side [65]. To avoid surgical damage, control rats received an injection of vehicle solution (2.5 µL 0.1 M PBS per side). Tibial fracture surgery was performed following the stereotaxic injection.

### 5.16 Lentivirus Transfection

To overexpress SIRT2 in HT22 cells, we employed lentivirus transfection technology. HT22 cells were transfected with Lentivirus5‐Sirt2 or Lentivirus5‐NC (GenePharma, Shanghai, China) according to relevant instructions.

### 5.17 Mitochondrial Superoxide Determination

HT22 cells were grown in six‐well glass‐bottom plates at a density of 2 × 10^4^/well. A 5 mM MitoSOX reagent stock solution (Thermo Fisher Scientific, M36008) was prepared using 2 mL of 5 µM MitoSOX reagent working solution. The cells adhered to coverslips were covered and incubated at 37°C for 10 min in the dark. Cells were then washed gently three times with a warm buffer and mounted in the warm buffer for imaging. MitoSOX was detected using a confocal fluorescence microscope (Axio Observer Z1; Zeiss, Oberkochen, Germany) at 510/580 nm excitation/emission.

### 5.18 Immunocytochemistry

HT22 cells were washed with PBS, fixed for 10 min (4% paraformaldehyde solution), and then washed three times with PBS for 15 min. The cells were then permeabilized for 5 min with 0.2% Triton X‐100 and blocked with 10% bovine serum albumin in PBS. The slides were incubated with anti‐PGC‐1α (1:100) and anti‐SIRT2 (1:100) overnight at 4°C. After washing three times, the cells were then incubated for 1 h with Alexa Fluor 488 (Abcam, ab150129) and Alexa Fluor CY3 (Jackson, 111‐165‐003) secondary antibodies at 37°C, and the nuclei were stained with DAPI for 8 min. Fluorescence images were obtained with a confocal fluorescence microscope.

### 5.19 Coimmunoprecipitation

The total protein in the cells was extracted and incubated with protein A/G‐coupled magnetic beads (Thermo Fisher Scientific, 20421) with PGC‐1α antibodies (Santa Cruz, sc‐518025) at 4°C overnight. Using Tris‐buffered saline buffer, the beads were washed four times and the supernatant was discarded, boiled at 95°C for 10 min, and eluted with sodium dodecyl sulfate loading buffer. Using antiacetylated lysine antibodies (CST, 9441S), immunoblot analysis was performed.

### 5.20 Statistical Analysis

The statistical analysis was conducted using GraphPad Prism 7 software (GraphPad Software Inc., La Jolla, CA, USA). The Shapiro–Wilk test was used to confirm the normality of the data distribution. Mean ± standard deviation is used to present quantitative data. Statistical significance was determined using analysis of variance followed by Bonferroni's post hoc test. The means of the two groups were compared using two‐way unpaired Student's *t*‐tests. *p* < 0.05 indicated statistical significance.

## Author Contributions

K.L., Z.L., and X.G. designed the study and wrote the manuscript. L.C., Q.W., Y.L., J.H., and Y.L. conducted the experimental operations. X.M. and Y.S. discussed the results. X.W. acquired and analyzed data. Y.Y. and J.W. provided technical support. D.H, T.L., and N.Y. revised the manuscript. All the authors have read and approved the final paper.

## Ethics Statement

Animal procedures were approved by the Animal Ethics Committee of Peking University Health Science Center (LA2021041).

## Conflicts of Interest

The authors declare no conflicts of interest.

## Supporting information



Supporting Information

## Data Availability

The datasets used and analyzed during the current study are available from the corresponding author upon reasonable request.
