# Supplementary material for: Swimming Exercise Pretreatment Attenuates Postoperative Delirium‐Like Behavior in Type 2 Diabetic Rats by Enhancing Mitochondrial Biogenesis Through Activation of SIRT2 Deacetylation
Source: MedComm (2020). 2025 Mar 18;6(4):e70142. doi: 10.1002/mco2.70142 (PMC11914775; doi:10.1002/mco2.70142)
Supplement: Supplementary file 1 — Supporting Information [file MCO2-6-e70142-s001.docx]

**Title:**

Swimming exercise pretreatment attenuates postoperative delirium-like behavior in type 2 diabetic rats by enhancing mitochondrial biogenesis through activation of SIRT2 deacetylation

**Authors:**

Kaixi Liu^1^, M.D.; Lei Chen^1^, Ph. D.; Xinning Mi^1^, M.D.; Qian Wang^1^, M.D.; Yitong Li^1^, Ph. D.; Jingshu Hong^1^, Ph. D.; Xiaoxiao Wang^2^, Ph. D.; Yue Li^1^, Ph. D.; Yanan Song^1^, M.D.; Yi Yuan^3^, M.D.; Jie Wang^4^, Ph.D.; Dengyang Han^1^, Ph.D.; Taotao Liu^1^, Ph.D.; Ning Yang^1^, Ph.D.; Xiangyang Guo^1,5,6^*, M.D., Ph.D.; Zhengqian Li^1,5,6^*, M.D., Ph.D.

1. Department of Anesthesiology, Peking University Third Hospital, Beijing 100191, China.
2. Research Center of Clinical Epidemiology, Peking University Third Hospital, Beijing, 100191, China.
3. Department of Anesthesiology, Beijing Jishuitan Hospital, Capital Medical University, Beijing 100035, China.
4. Department of Anesthesiology and Perioperative Medicine. People's Hospital of Zhengzhou University, Zhengzhou 450003, China
5. Executive Office, Beijing Center of Quality Control and Improvement on Clinical Anesthesia, Beijing,100191, China.
6. Perioperative Medicine Branch of China International Exchange and Promotive Association for Medical and Health Care (CPAM), Beijing 100191, China.

**Address for Correspondence:**

*Authors to whom correspondence should be addressed at the Department of Anesthesiology, Peking University Third Hospital, No.49 North Garden Rd, Haidian District, Beijing, 100191, PR China. E-mail address: zhengqianli@hsc.pku.edu.cn to ZQ Li, puthmazk@hsc.pku.edu.cn to XY Guo.


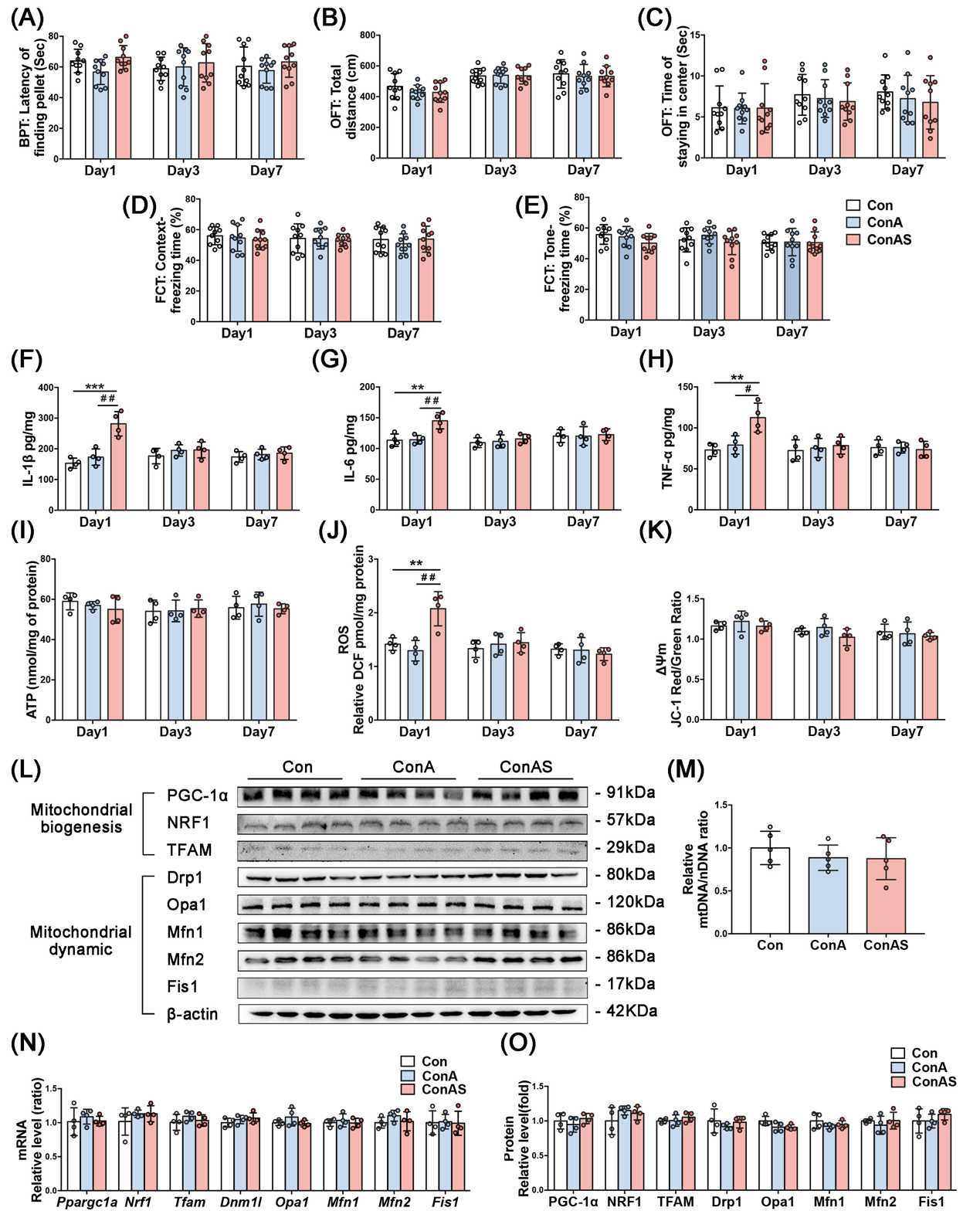


**Figure S1. Effect of anesthesia or surgery on postoperative neurobehavior, neuroinflammatory response and mitochondrial function in normal adult rats.** (A) Normal rats receive BPT on postoperative days 1, 3, and 7. (B) Total distance and (C) the time of staying in the center of normal rats in OFT after anesthesia or tibial fracture surgery. (D) Freezing time of context test and (E) tone test of normal rats in FCT after anesthesia or tibial fracture surgery. (F–H) Expression levels of pro-inflammatory factors in hippocampal tissue of normal rats. (I–K) Effect of anesthesia or surgery on ATP, ROS, and MMP levels in the hippocampus of normal rats. (L) Representative bands of mitochondrial biogenesis and dynamics-related molecules in the hippocampus. (M) Relative levels of mtDNA/nDNA. (N) Relative mRNA expression levels (O) and statistical analyses of mitochondrial biogenesis and dynamics-related molecules in the hippocampus. *P < 0.05, **P < 0.01, ***P < 0.001 compared with Con group. ^#^ P < 0.05, ^##^ P < 0.01, ^###^P < 0.001 compared with ConA group.


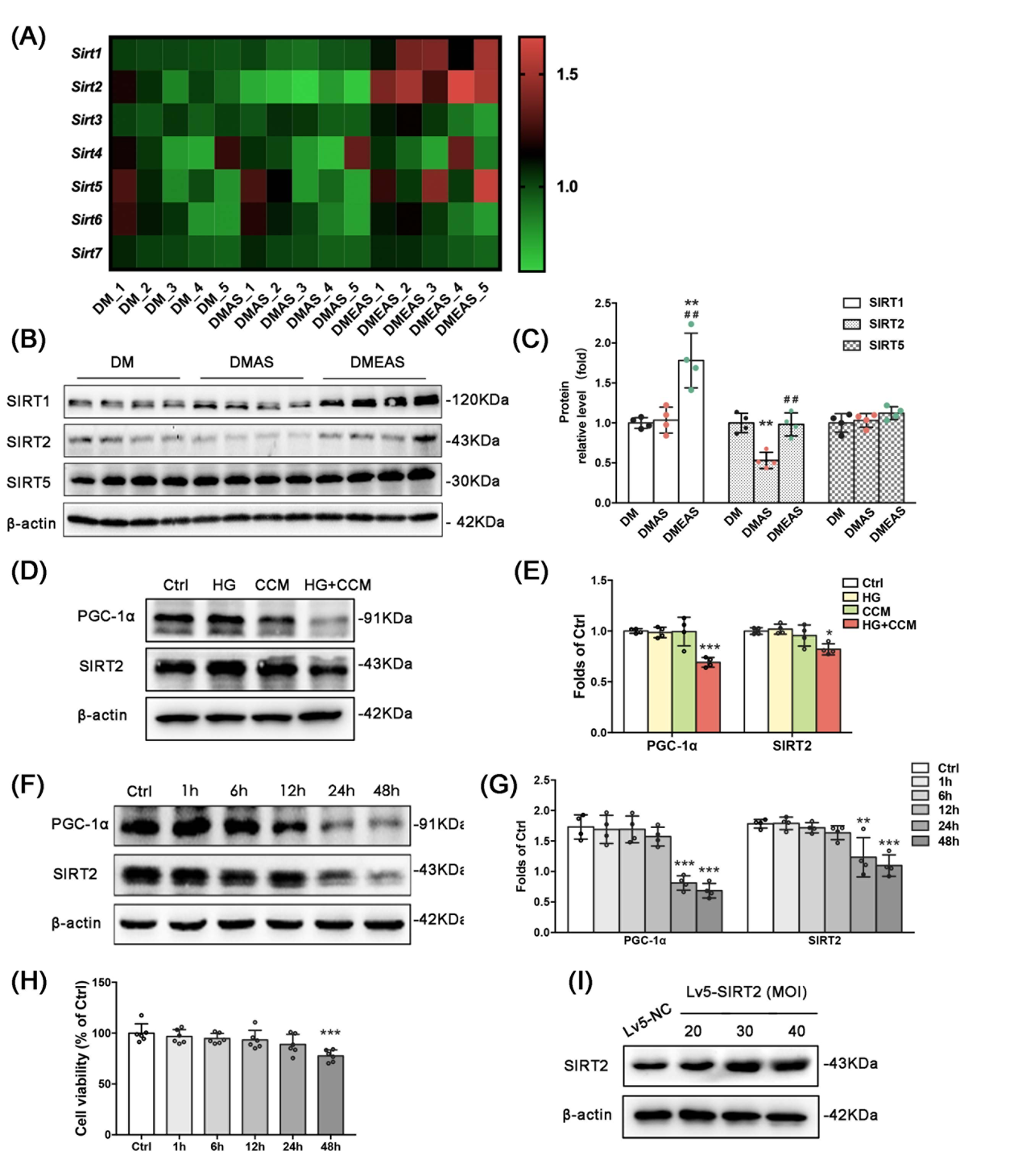


**Figure S2**. **Screening of conditions for in vivo and in vitro experiments.** (A) Relative mRNA expression levels of *Sirt1-7.* (B) Representative bands and (C) statistical analyses of SIRT1, 2, 5 in the hippocampus. (D) Representative bands and (E) statistical analyses of the protein expression levels of PGC-1 α and SIRT2 expression in HT22 cells under different conditions for 24 h. (F) Representative bands and (G) statistical analyses of the protein expression levels of PGC-1 α and SIRT2 expression in HT22 cells treated with HG+CCM with different time gradients. (H) CCK8 detected the activity of HT22 cells treated with HG+CCM with different time gradients. (I) Protein expression levels of SIRT2 in HT22 cells transfected with Lv5-NC or Lv5-SIRT2 with various MOI (20, 30, and 40) for 72 h.

**Supplementary 1**

Sirtuins 1–7 are proteins that belong to the third class of deacetylase enzymes, which are dependent on NAD^+^ for activity. Deacetylation is associated with the enhancement of mitochondrial ATP production capacity. Sirtuin activity is linked to gene repression, metabolic control, inflammation, and neuroprotection. We examined the mRNA levels of sirtuin proteins in each group and found that *Sirt1, Sirt2,* and *Sirt5* were significantly upregulated in the DMEAS group compared with DM and DMAS groups. However, the levels of *Sirt1* and *Sirt5* in the DMAS group did not show a significant reduction compared with the DM group (Figure S2. A). We focused on SIRT2, which was decreased after surgical stress and upregulated by exercise. Further, the results of Western blot analysis were consistent with the PCR results (Figure S2. B, C).

**Supplementary 2**

On the basis of our *in vivo* animal experiments, to further validate the role of SIRT2 in cell models, the HT22 neuronal cell line was selected. Bone marrow cells from the femur and tibia of 8-week-old C57BL/6 mice were obtained, and bone marrow-derived macrophages (BMDMs) were then obtained after macrophage colony-stimulating factor cultured for 7 days, followed with 10 ng/ml lipopolysaccharide (LPS) stimulated for 2 h. After replacing the complete medium and culturing for 24 h, the conditioned culture media (CCM) was collected. D-glucose was then added to obtain 50 mM of high-sugar CCM to stimulate the HT22 cells and mimic the effects of high-sugar, inflammatory conditions on neurons (Figure 6. A).

To further investigate the effects of the high-glucose, inflammatory conditions on SIRT2 and mitochondrial biogenesis, normal medium (Ctrl), 50 mM high glucose-alone (HG), conditioned medium-alone (CCM), and high glucose plus conditioned medium (HG+CCM) groups were devised. WB showed that, compared with the Ctrl group, the HG+CCM group had significantly lower PGC-1α and SIRT2 expression levels, while the expression of PGC-1α and SIRT2 was not significantly different between the Ctrl group and the HG and CCM groups (Figure S2 D, E). Further, the CCK8 assay showed that HT22 cell viability was significantly reduced at 48 h in the HG+CCM group compared with the Ctrl group, and the expression of SIRT2 and PGC-1α exhibited significant downregulation at 24 and 48 h (Figure S2. F–H). Therefore, the time point of 24 h, at which no significant change in cell viability was detected, was used in the subsequent experiments. Lentivirus Lv5-SIRT2 was used to achieve overexpression of SIRT2. First, Lv5-SIRT2 was transfected into HT22 cells for 72 h to determine the efficiency of viral transfection, and the WB results showed that the efficiency of transfection was sufficient, at 180%–240%, when the multiplicity of infection was 30 (Figure S2. I).

**Supplementary 3**

The list of primers

| **mRNA** | **Sequence** |
| --- | --- |
| *Ppargc1a-F* | 5′-AAAGGATGCGCTCTCGTTCG-3′ |
| *Ppargc1a-R* | 5′- GTGTGCGGTGTCTGTAGTGG-3′ |
| *Nrf1-F* | 5′-TCTGCTGTGGCTGATGGAGAGG-3′ |
| *Nrf1-R* | 5′-GATGCTTGCGTCGTCTGGATGG-3′ |
| *Tfam-F* | 5′-TAGAGAAGGAAGCCCGGCAG-3′ |
| *Tfam-R* | 5′-GCTGACTCATCCTTAGCCTCCT-3′ |
| *Dnm1l-F* | 5′-GAGAACTACCTTCCGCTGTATCGC-3′ |
| *Dnm1l-R* | 5′-CACCATCTCCAATTCCACCACCTG-3′ |
| *Opa1-F* | 5′-ATGCTCGCTATCACTGCCAACAC-3′ |
| *Opa1-R* | 5′-CCTTCTTCTCGCCGTCTTCAGC-3′ |
| *Mfn1-F* | 5′-CGTGGCAGCAGCAGAGAAGAG-3′ |
| *Mfn1-R* | 5′-CCTCCTCCGTGACCTCCTTGATC-3′ |
| *Mfn2-F* | 5′-TCCACAGCCATTGCCAGTTCAC-3′ |
| *Mfn2-R* | 5′-CCGCACAGACACAGGAAGAAGG-3′ |
| *Fis1-F* | 5′-GAATACGCCTGGTGCCTGGTTC-3′ |
| *Fis1-R* | 5′-GAAGACATAATCCCGCTGCTCCTC-3′ |
| *Actb-F* | 5′-CGTTGACATCCGTAAAGACCTC-3′ |
| *Actb-R* | 5′-TAGGAGCCAGGGCAGTAATCT-3′ |
| **DNA** | **Sequence** |
| *Rat-mtDNA-F* | 5′-CAAACCTTTCCTGCACCTCC-3′ |
| *Rat-mtDNA-R* | 5′-AGGCGTTCTGATGATGGGAA-3′ |
| *Mouse-mtDNA-F* | 5′-CTAGCAGAAACAAACCGGGC-3′ |
| *Mouse-mtDNA-R* | 5′-CCGGCTGCGTATTCTACGTT-3′ |
| *Rat-nDNA-F* | 5′-GTTCCCGCCTTCTTCCTCTG-3′ |
| *Rat-nDNA-R* | 5-GTTTGCTTGCCGACTCCTTG-3′ |
| *Mouse-nDNA-F* | 5′- GCCAGCCTCTCCTGATTTTAGTGT-3′ |
| *Mouse-nDNA-R* | 5′-GGGAACACAAAAGACCTCTTCTGG-3′ |
